# Optimal estimated standard uncertainties of reflection intensities for kinematical refinement from 3D electron diffraction data

**DOI:** 10.1107/S2053273323005053

**Published:** 2023-08-14

**Authors:** Malak Khouchen, Paul Benjamin Klar, Hrushikesh Chintakindi, Ashwin Suresh, Lukas Palatinus

**Affiliations:** a Institute of Physics of the Czech Academy of Sciences, Prague, Czech Republic; b University of Bremen, Bremen, Germany; University of Warsaw, Poland

**Keywords:** error modelling, error analysis, data reduction, electron diffraction

## Abstract

Estimating the error in the merged reflection intensities requires a full understanding of all the possible sources of error arising from the measurements. Most diffraction-spot integration methods focus mainly on errors arising from counting statistics for the estimation of uncertainties associated with the reflection intensities. This treatment may be incomplete and partly inadequate. In an attempt to fully understand and identify all the contributions to these errors, three methods are examined for the correction of estimated errors of reflection intensities in electron diffraction data. For a direct comparison, the three methods are applied to a set of organic and inorganic test cases. It is demonstrated that applying the corrections of a specific model that include terms dependent on the original uncertainty and the largest intensity of the symmetry-related reflections improves the overall structure quality of the given data set and improves the final *R*_all_ factor. This error model is implemented in the data reduction software *PETS2*.

## Abstract 2

Several models for estimating the standard uncertainties of reflection intensities are analysed for refinement against 3D electron diffraction data. A new model is proposed which results in more accurate structure models.

## 1. Introduction

The use of electron diffraction (ED) for crystal structure determination has grown rapidly over the past decade, particularly thanks to the introduction of 3D methods for the systematic acquisition and analysis of diffracted intensities. 3D ED techniques have been shown to be powerful for structure determination of crystals that are too small for single-crystal X-ray diffraction analysis. These techniques benefit from the strong Coulomb interaction between electrons and matter. This allows 3D single-crystal ED to be obtained from nanocrystals that are about eight orders of magnitude smaller in volume than those needed for single-crystal X-ray diffraction. Several 3D ED techniques, which share the common concept of tilting a crystal around the goniometer axis and acquiring a series of ED patterns, have been presented and developed over the years. These include automated diffraction tomography (ADT) (Kolb *et al.*, 2007 ▸), rotation electron diffraction (RED) (Zhang *et al.*, 2010 ▸) and precession electron diffraction tomography (PEDT) (Mugnaioli *et al.*, 2009 ▸). Since the rise of sensitive detectors with negligible readout time, continuous-rotation 3D ED has become the most popular protocol for data acquisition (Nederlof *et al.*, 2013 ▸; Nannenga *et al.*, 2014 ▸; Wang *et al.*, 2017 ▸). Several software suites are available nowadays for 3D ED data reduction, thus allowing a 3D visualization of the data set, the determination of cell parameters, reconstruction of the 3D reciprocal lattice and extraction of reflection intensities with their estimated standard uncertainties (e.s.u.’s). Determining the e.s.u.’s of the reflection intensities is challenging. Error estimates of reflection intensities from electron-counting statistics alone may underestimate the real uncertainty associated with the measurements. The accurate estimation of e.s.u.’s is important throughout the process of crystallographic structure analysis. The result of structure refinement and the accuracy of the refined parameters depend on the correct estimation of the e.s.u.’s.

The determination of reflection e.s.u.’s is an important part of the data reduction process. It has turned out that pure counting-statistics-based estimates are not optimal, and other effects must be included in the determination of e.s.u.’s. The model describing the adjustment of e.s.u.’s is called the error model. The practice of refining the error model and correcting for various effects has a long history (Diamond, 1969 ▸; Abrahams & Keve, 1971 ▸; Rossmann *et al.*, 1979 ▸; Schwarzenbach *et al.*, 1989 ▸; Howell & Smith, 1992 ▸; Leslie, 1999 ▸; Evans, 2006 ▸, 2011 ▸).

This paper aims to analyse methods of treating the error estimates of the integrated intensities from 3D ED data for the purpose of kinematical refinement and indicate the best error model. All the structures studied in this paper are processed using *PETS2* (Palatinus *et al.*, 2019 ▸) and refined using *JANA2020* (Petříček *et al.*, 2023 ▸), but the general ideas also apply to other implementations of 3D ED data reduction software.

## 2. Experimental, data processing and refinement setup

Throughout the text we use three data sets to assess and validate the presented methods. The materials are the natural zeolite natrolite, (*S*)-(+)-ibuprofen and the amino acid l-alanine.

### 2.1. Natrolite

Continuous-rotation 3D ED experiments were performed at different spots of the selected crystal which showed signs of mosaicity of about 0.15°. The crystal diffracted up to a resolution d* of about 1.6 Å^−1^ at a temperature of 293 K (see Tables 1 ▸ and 2 ▸).

A centre of symmetry was added in the averaging process in *JANA2020*. The default weighting scheme with weights was 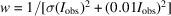 and an extinction correction was applied in the refinement. The model was refined against 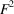 and against all reflections. The geometry of the water molecule was restrained to distances of 0.9584 Å between H atoms and their relative O atom and to an angle of 104.45°. All non-H atoms were refined with anisotropic displacement parameters (ADPs). A riding model was used for the ADPs of H atoms, with an extension factor of 1.2. Reference covalent bond lengths of non-H atoms for the calculation of the root-mean-square deviation (RMSD) were taken from a single-crystal X-ray diffraction (XRD) study on natrolite (Capitelli & Derebe, 2007 ▸).

### 2.2. (*S*)-(+)-ibuprofen

Continuous-rotation 3D ED experiments were performed at different spots of two selected crystals which showed signs of mosaicity of 0.135°. Two data sets were collected and then merged. The crystals diffracted up to a resolution of about 1 Å^−1^ at a temperature of *T* = 95.15 K (see Tables 3 ▸ and 4 ▸).

A centre of symmetry was added in the averaging process in *JANA2020*. The default weighting scheme with weights was 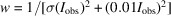 and an extinction correction was applied in the refinement. The model was refined against 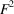 and against all reflections. H atoms bonded to carbon were placed in idealized positions. H atoms bonded to oxygen were restrained at distances of 0.98 Å away from their relative O atom and the COH angle of molecule *A* was restrained to be equal to its corresponding analogue in molecule *B*.

XRD-based reference atomic distances for the calculation of RMSD were taken from King *et al.* (2011 ▸).

### 2.3. l-alanine

Continuous-rotation 3D ED experiments were performed at different spots of a selected crystal which showed signs of mosaicity of 0.3°. One data set was collected and used. The crystal diffracted up to a resolution of about 2.0 Å^−1^ at a temperature of *T* = 100 K (see Tables 5 ▸ and 6 ▸).

A centre of symmetry was added in *JANA2020* in the averaging process. The default weighting scheme with weights was 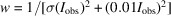 and an extinction correction was applied in the refinement. The model was refined against 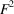 and against all reflections. All non-H atoms were refined with ADPs. A riding model was used for the ADPs of H atoms, with an extension factor of 1.2.

XRD-based reference atomic distances for the calculation of RMSDs were taken from Parsons *et al.* (2013 ▸).

## 3. Methods of adjustment of error estimates

In this section, we examine three approaches for the correction of error estimates. In *PETS2*, the integral intensity of a reflection on a single diffraction pattern is calculated as

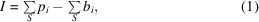

where 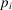 is the detector count in pixel *i* in the peak area *S* and 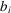 is the estimated background value for the same pixels. The summation runs over a region *S*. The background values are estimated from the detector count in the rim around the peak region *S*: 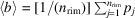 , where *n*_rim_ is the number of pixels in the rim. The total estimated background in the region *S* is given by 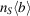 and the background-corrected integrated intensity can then be expressed as

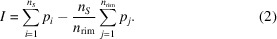




*PETS2* employs the following formula to calculate the e.s.u. of each pixel (Waterman & Evans, 2010 ▸):

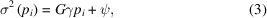

where *G*, γ and 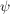 are the noise parameters characterizing each detector. γ is a ‘cascade factor’, accounting for the intensity-dependent increase of variance above the Poisson-statistics value, and 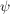 is a ‘pixel factor’ which corresponds to the variance of pixel values of a dark image. *G* is the gain factor of the detector used that converts the number of incident electrons to the number of counts in the digitized diffraction image (detector-readout values).

Then, using the propagation-of-errors method, the variance of the integrated intensity can be expressed as

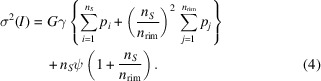

This represents the variance in the integrated intensity, taking into account the Poisson noise and detector-related increase of the variance. It is crucial to have reasonable values of *G*, γ and 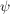 to obtain accurate error estimates (Waterman & Evans, 2010 ▸).

However, these Poisson-based standard deviations underestimate the true e.s.u.’s, and additional adjustment needs to be made to these e.s.u.’s to address any additional uncertainty introduced by other sources of errors than the Poisson noise and detector, such as instrumental instability. One way to deal with these errors is to inflate the error estimates by adding extra terms that account for the additional uncertainty.

### 3.1. Methodology

The starting idea is based on the analysis of intensity distribution of multiply measured and symmetry-equivalent reflections. In an ideal data set, these reflections should have identical intensities. In practice, this is not the case due to, for example, statistical noise and non-kinematical scattering. The e.s.u.’s of individual reflections should thus properly characterize any deviation from the expected equality of multiply measured reflections. Based on this concept, various methods can be devised that produce better standard uncertainty estimates. Using such a method has become a *de facto* standard in modern data-processing software. However, to our knowledge, its validity for 3D ED data has not yet been investigated. There are at least two reasons why it cannot be automatically assumed that the method developed for X-ray diffraction data is also valid for 3D ED data. First, the nature of errors in the 3D ED data is different. The most significant deviations from the ideal kinematical intensities do not arise from random errors and instrumental effects, but from the dynamical diffraction effects. These effects are unavoidable in 3D ED data. Strictly speaking, these effects are not sources of errors in the data, and they should be modelled as part of the calculation of calculated intensities in the refinement process (Palatinus, Petříček & Corrêa, 2015 ▸; Palatinus, Correa *et al.*, 2015 ▸). However, when kinematical approximation is used in the refinement, these effects effectively turn into errors in reflection intensities which, although not random, may be reflected in the values of e.s.u.’s. The second reason for special consideration is that, compared with X-ray diffraction data, the spread of the equivalent intensities around the mean value is typically much larger than in X-ray data. This is again caused mainly by the dynamical diffraction, but also often by radiation damage of the crystal, and it is reflected in increased values of *R*_int_, which frequently reach 20% or more (Bruhn *et al.*, 2021 ▸). This large spread may lead to larger corrections to e.s.u.’s and may induce effects that are not significant in X-ray diffraction data. The analysis in this paper shows this is indeed the case.

In the following, we analyse and compare three models for adjusting the e.s.u.’s of three different 3D ED experimental data sets. The efficiency of each method is assessed in a number of ways. We first evaluate the quality of each error model by checking the normality of the obtained distribution of residuals. A kinematical refinement is then performed and the refinement figures of merit are compared. The assessment is also based on comparing the RMSD of refined covalent bond lengths and the RMSD of atomic shifts for all non-H atoms with reference structures.

### 3.2. Model 0: no adjustment

For comparison purposes, the model with no adjustment to e.s.u.’s is also included in the analysis and it is denoted model 0. In this model, the e.s.u.’s are calculated using equation (4) without further modifications.

### 3.3. Model 1: using equivalent errors

In the first adjustment model, the reflection e.s.u.’s are calculated from the variation of symmetry-related intensities around their mean. The e.s.u. is calculated as a sample standard deviation from the *n* measurements of the symmetry-equivalent reflections. Given a reflection index **h** with *n* measurements of the intensity of **h** or its symmetry equivalent, we define the *l*th measurement of **h** as 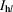 . All 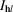 are associated with a common e.s.u. 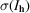 defined as

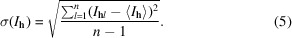

The division by 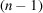 instead of *n* is known as Bessel’s correction, and it corrects for the bias in the estimation of the population standard deviation from only the sample of the population. The mean of all the symmetry-related measurements is used to estimate the reflection intensity 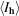 :

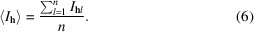




**Figure 1:**
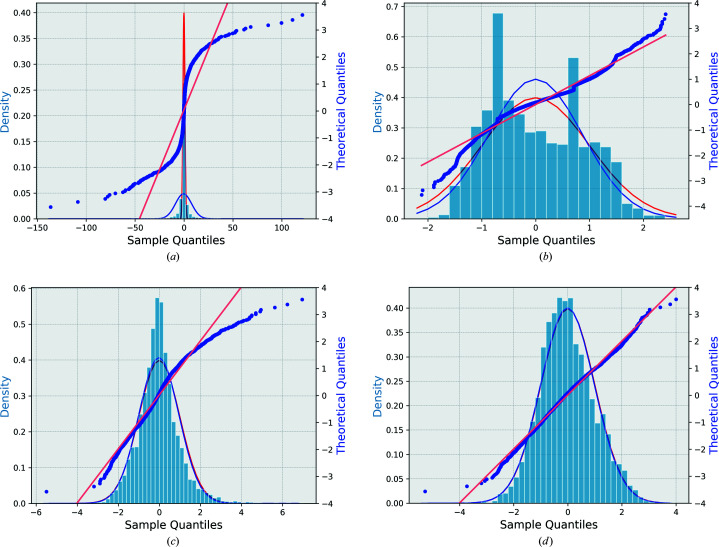
Distribution of the normalized deviations of each model (blue histogram) versus the normalized Gaussian distribution (in red) for natrolite. The blue curve is the best-fit Gaussian distribution to the histogram. The normal probability plot (blue dotted line) represents the normalized deviations versus the theoretical quantiles of natrolite for all the data with *N* = 5362 reflections (not including eight individual single reflections having zero delta). The straight line is the best-fit line to the normal probability plot. (*a*) Original model normalized deviations and normal probability plot of original uncorrected error estimates data (model 0). (*b*) Adjusted normalized deviations and normal probability plot of model 1. (*c*) Adjusted normalized deviations and normal probability plot of model 2. (*d*) Adjusted normalized deviations and normal probability plot of model 3.

**Figure 2:**
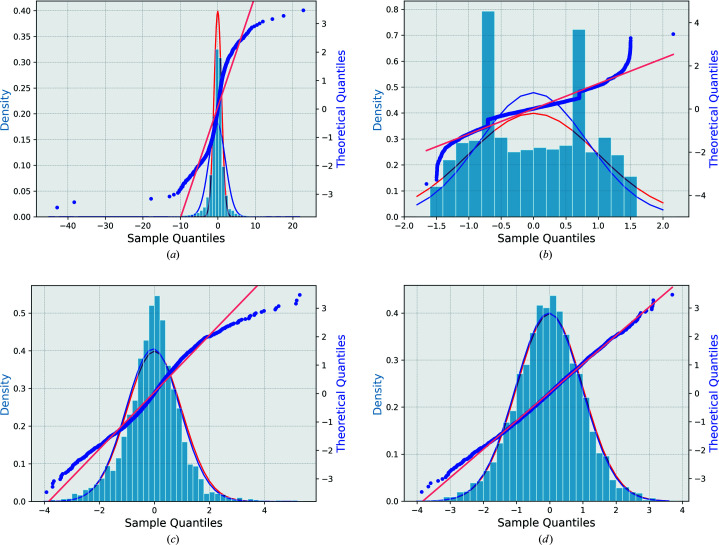
Distribution of the normalized deviations of each model (blue histogram) versus the normalized Gaussian distribution (in red) for (*S*)-(+)-ibuprofen. The blue curve is the best-fit Gaussian distribution to the histogram. The normal probability plot (blue dotted line) represents the normalized deviations versus the theoretical quantiles of (*S*)-(+)-ibuprofen for all the data with *N* = 3855 reflections (not including 37 single reflections having zero delta). The straight line is the best-fit line to the normal probability plot. (*a*) Original model normalized deviations and normal probability plot of original uncorrected error estimates data (model 0). (*b*) Adjusted normalized deviations and normal probability plot of model 1. (*c*) Adjusted normalized deviations and normal probability plot of model 2. (*d*) Adjusted normalized deviations and normal probability plot of model 3.

**Figure 3:**
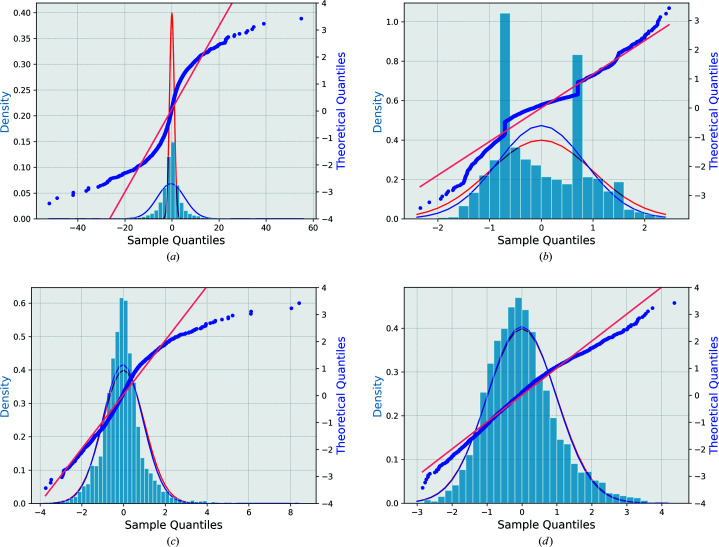
Distribution of the normalized deviations of each model (blue histogram) versus the normalized Gaussian distribution (in red) for l-alanine. The blue curve is the best-fit Gaussian distribution to the histogram. The normal probability plot (blue dotted line) represents the normalized deviations versus the theoretical quantiles of l-alanine for all the data with *N* = 3255 reflections (not including 197 single reflections having zero delta). The straight line is the best-fit line to the normal probability plot. (*a*) Original model normalized deviations and normal probability plot of original uncorrected error estimates data (model 0). (*b*) Adjusted normalized deviations and normal probability plot of model 1. (*c*) Adjusted normalized deviations and normal probability plot of model 2. (*d*) Adjusted normalized deviations and normal probability plot of model 3.

This model does not use the original Poisson counting error estimates and assumes that a large enough sample of equivalent reflections is available to reliably estimate the uncertainty. This assumption is often not well fulfilled because 3D ED data sets from low-symmetry crystals, in particular, exhibit a limited completeness and redundancy. In extreme cases, a number of reflections may be measured only once (*n* = 1), and their e.s.u.’s cannot be calculated using the above relation. To include these reflections in the structure refinement, we construct a lookup table which allows estimation of the e.s.u.’s of those reflections from the e.s.u.’s of other reflections with similar resolution and intensity. Firstly, we sort the reflections in order of increasing intensity and then we divide them into ten intensity bins with 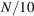 reflections in each bin (*N* is the total number of reflections). The second binning divides the reflections into resolution bins with a bin width of 0.2 Å^−1^. Then the average of e.s.u.’s of all reflections in the same bin is calculated and assigned as the e.s.u. of all individually measured reflections in the same bin. The residuals 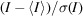 in the original data (model 0) clearly deviate from a normal distribution as shown in Fig. 1 ▸(*a*) for natrolite, Fig. 2 ▸(*a*) for (*S*)-(+)-ibuprofen and Fig. 3 ▸(*a*) for l-alanine. Figs. 1 ▸(*b*), 2 ▸(*b*), 3 ▸(*b*) show the analogous figures corresponding to the adjusted data according to model 1. The straight horizontal segments (constant steps) in the normal probability plot of this model at the sample quantile of −0.707 and 0.707, and the peaks in the histograms of normalized deviations at ±0.707 are due to reflections measured twice (*n* = 2), as follows from equations (5), (6) and (8) for the special case of *n* = 2. This feature and the general mismatch between the distribution of the residuals and the expected normal distribution show that model 1 is not optimal for low-multiplicity data sets. The analysis of the structure refinements shows (Section 4.1) that this model leads to a worse (ibuprofen and l-alanine) or only marginally better (natrolite) structure model than model 0.

### 3.4. Model 2: three-parameter model using average intensities of symmetry-related reflections

This model was first introduced by Evans (2006 ▸). It uses the normal probability plot (Abrahams & Keve, 1971 ▸) to adjust the error estimates of the integrated intensities. The model introduces a small number (two or three) of correction parameters that are used to modify the e.s.u.’s. The correction factors are optimized to make the normal probability plot as linear as possible. In this paper, we follow the notations of the three correction parameters from Brewster *et al.* (2019 ▸). The corrected error estimates are given by

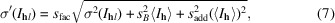

with 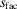 , 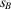 and 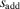 being the correction parameters. Two of the three parameters have a physical interpretation. According to Evans (2011 ▸), 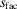 is understood as a correction factor for unknown errors independent of the intensity value including uncertainty in the detector gain used to estimate Poissonian errors. It acts like a scaling factor. 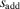 is a parameter that accounts for any errors that are proportional to the intensity such as instrument instabilities. 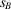 has no direct physical meaning and it is excluded in some programs [such as *XDS* (Kabsch, 2010 ▸)], but it is an obvious addition to the parameter set, and in this work we include it to provide maximum flexibility of the fitting.

#### 3.4.1. Normalized deviation

Estimates of error such as 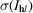 represent the statistically expected deviation of the measurement’s intensity 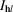 from the unknown population mean value. Assuming a Gaussian distribution of the intensity errors, the normalized deviations of the measurements 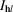 from the mean value of the symmetry-related reflections 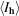 are expected to be distributed according to a standard normal distribution. The normalized deviations 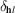 for 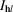 are given by

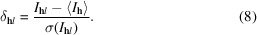




According to Evans (2006 ▸), 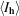 is the mean of the measurements of **h** excluding the *l*th reflection 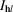 . In this work, we consider 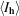 as the average intensity over all observations of reflection **h** including the *l*th reflection:

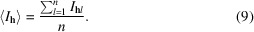

This means that for reflections measured only once, where 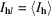 , the corresponding normalized deviation will be 0. This choice affects mainly the refinement of the 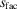 parameter. In the approach used by Evans (2006 ▸), excluding a strong reflection from the calculation of 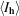 , the resulting average intensity is reduced. The normalized deviation will consequently get larger, leading to larger error estimates, higher 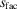 and consequently fewer observed reflections (see Section 5.1 for discussion of the meaning of observed reflections in 3D ED data). When all reflections are included in the calculation of the average, the 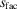 parameter is reduced, leading to a larger number of observed reflections. In the work of Evans (2006 ▸), the average intensity 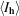 is also a weighted average, where the weights are given by inverse variance estimates of the individual observations. This approach biases the average intensity towards the weaker reflections, which have, in general, lower e.s.u.’s and hence higher weights, leading to the same effects as excluding strong reflections from the average, discussed above. We also tested this approach and concluded that the non-weighted average 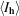 gives better results.

The aim of the method is adjustment of the parameters 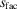 , 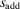 and 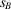 to make the distribution of the normalized deviations as close as possible to the standard normal distribution, *i.e.* a Gaussian distribution centred on zero with a standard deviation of 1.

#### 3.4.2. Normal probability plot technique

The values of the correction parameters 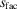 , 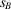 and 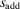 can be conveniently determined by optimizing the normal probability plot (Abrahams & Keve, 1971 ▸). The normal probability plot is a plot of the sorted normalized deviations 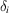 versus the perfectly distributed quantiles 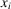 expected for a normal distribution. The values of 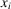 are known as the theoretical quantiles and they correspond to a normal distribution of zero mean and standard deviation of 1. A normal probability plot of a variable with perfect standard normal distribution is a line with intercept 0 and slope 1. To find the matching normal distribution quantiles, we first calculate the cumulative distribution function (CDF) of the standard normal distribution. It is usually denoted by 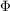 and has the general form

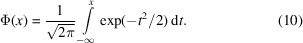

The *i*th element of a sorted sample with standard normal distribution represents the value with 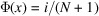 . Conversely, the inverse of the CDF 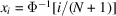 gives the expected value of the *i*th element of a sorted sample, *i.e.* the so-called theoretical quantile of the standard normal distribution.

The normal probability plot thus contains *N* points with coordinates 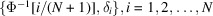 , where 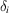 is the *i*th normalized deviation in the list sorted from the smallest to the largest normalized deviation. The individual reflections having 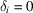 are not included in the list. Figs. 1 ▸, 2 ▸ and 3 ▸ show normal probability plots for different error models for all three experimental data sets. The values of 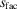 , 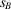 and 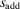 can be adjusted to make the normal probability plot as close to the ideal straight line with slope 1 as possible.

#### 3.4.3. Initial parameters

Evans (2006 ▸) proposed obtaining the initial value of 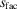 by fitting the slope of the central part of the normal probability plot in the theoretical quantiles range between −0.5 and 0.5. However, we observed that the least-squares fitting procedure is so robust that good convergence is obtained even if the fit starts from the default values 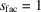 , 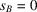 and 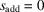 .

#### 3.4.4. Corrected error estimates

Using equation (7) for 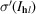 , we calculate the adjusted error estimates using the current set of correction parameters. Then, the new normalized deviations 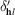 are computed using

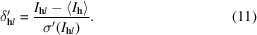




These normalized deviations are then sorted, and a new normal plot is calculated. To obtain optimal values of the correction parameters, we minimize the quantity 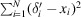 , which designates the sum of the squared difference between the adjusted normalized deviations and the theoretical quantiles. Note that, after one minimization, the normalized deviations must be resorted, as their order may change.

After sorting, a new minimization must be performed, and the supercycle repeated until convergence. As the number of data points is large and the number of fitted parameters is small, the convergence is usually rapid and robust. Figs. 1(*a*) ▸, 2(*a*) ▸ and 3(*a*) ▸ show the original and Figs. 1(*c*) ▸, 2(*c*) ▸ and 3(*c*) ▸ the optimized normal probability plots and the histograms of the adjusted normalized deviations and their comparison with the standard normal distribution of natrolite, (*S*)-(+)-ibuprofen and l-alanine, respectively.

### 3.5. Model 3: three-parameter model using the largest intensities of symmetry-related reflections

Model 2 provides a clear improvement in comparison with model 1 or with unmodified e.s.u.’s. However, upon closer inspection, this model suffers from certain inadequacies. As shown in Figs. 1 ▸(*c*), 2 ▸(*c*) and 3 ▸(*c*), the normal probability plots representing the adjusted normalized deviations versus the theoretical quantiles in the case of model 2 do not match the line of slope 1, especially at the tails. By investigating the individual cases, we realized that model 2 provides poor results, especially for reflections that exhibit a considerable variation among the intensities of the symmetry-related reflections, *i.e.* if the variation is large compared with the intensity value itself. Upon optimization of the three error-model parameters, this model tries to compensate for this variation by assigning very large error estimates to very strong reflections. This leads to, among other effects, a notable reduction in the number of observed reflections. This problem is not very severe for typical X-ray diffraction data, where the symmetry-related reflections tend to have very similar intensities, but it is significant in 3D ED data, where the intensity variation can be very large. To correct this problem, we propose a new model (model 3), which is very similar to model 2 except that in this correction method we use the largest intensity 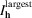 of the symmetry-related reflections for the calculations of the corrected error estimates rather than the average intensity 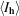 . The corrected error estimates are thus given by

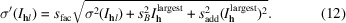

Here 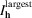 is the largest intensity of all the symmetry-related reflections of reflection **h**. The procedure for the optimization of the model parameters is the same as in model 2. The effect of this change is the decrease of the fitted values *s*_fac_, *s_B_* and *s*_add_. For reflection groups with little variation around 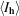 this means a smaller 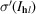 , because the error-model parameters are smaller and 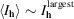 . However, for reflection groups with large variation, the smaller values of the error-model parameters are compensated by replacing the (smaller) 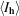 by the larger 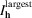 . The net result is then a relative increase of the 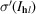 of reflection groups with a large variation compared with the reflection groups with a small variation.

Figs. 1 ▸(*d*), 2 ▸(*d*) and 3 ▸(*d*) show the final normal probability plots for this model. These can be compared with the other models. There is a pronounced improvement in the fitting of the normalized deviations of the error estimates. The improvement is also visible in the distribution of the normalized deviations of this model which shows that the adjusted normalized deviations are more accurately normally distributed in model 3 than in any other model including model 2.

#### 3.5.1. Outlier rejection

There is usually a group of errors that do not match the statistical distribution. They are most frequently caused by unpredictable experimental effects that cannot be corrected for. Measurements affected by such errors are known as outliers (Blessing, 1997 ▸). It is advantageous to exclude such outliers from the final data set, as they most likely indicate an erroneous measurement that cannot be properly fitted by the structure model.

We tested a number of outlier rejection algorithms, including the algorithm used in *SCALA* (or *AIMLESS*) (Evans, 2006 ▸). Finally, we converged to the use of Tukey’s simple but very robust rule of thumb that is based on the quartiles of the given data set (Tukey, 1977 ▸): firstly, calculate the first quartile 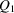 of 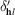 (25% of the normalized residuals are less than or equal to this value) and the third quartile 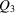 (25% of the 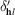 values are greater than or equal to this value). Outliers are then defined as all values that fall outside the range

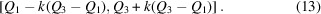

Tukey proposed using 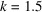 to identify outliers.

As the exclusion of outliers has an impact on the normal probability plot and hence on the refined error model, an iterative procedure needs to be adopted. Firstly, consider the original data set before applying any corrections to the e.s.u.’s and apply Tukey’s outlier rejection procedure as described above. In the case of only two equivalent reflections, if one is marked as an outlier, the other is marked too. Then, apply the error-model refinement to calculate the values of the correction parameters *s*_fac_, *s_B_* and *s*_add_, excluding the outliers from the calculation. Use the obtained values to correct the e.s.u.’s of all the reflections including the outliers. Apply Tukey’s outlier rejection again to the new data set with adjusted e.s.u.’s. Iterate the error-model refinement and outlier rejection until the values of the correction parameters *s*_fac_, *s_B_* and *s*_add_ converge and the number of identified outliers does not change. In the tests presented in this paper, the value of 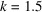 proved to be an appropriate value. However, in specific cases, this parameter may be adjusted to increase or reduce the number of outliers, if needed.

Kinematical refinement was carried out on each of the three data sets with outliers rejected to demonstrate the potential impact of the above-described outlier rejection approach, using model 3. The algorithm rejected 35, 66 and 93 outliers in the data of natrolite, ibuprofen and l-alanine, respectively, representing 0.65%, 1.7% and 2.69% of all measured reflections. The kinematical refinement results after applying the above outlier rejection algorithm are shown in Table 10.

## 4. Results

To assess the efficiency of each of the models presented above, we apply the above error correction models to data from three materials, as described in Section 2. Each data set was processed using the software *PETS2*. All structures could be solved from the data by the charge-flipping algorithm as implemented in *Superflip* (Palatinus & Chapuis, 2007 ▸) and refinements within the kinematical approximation were performed in *JANA2020*. Input data from models 0, 1, 2 and 3 were subject to refinement using the same data processing procedure, the same starting structure model, the same refinement parameters *etc*. The only difference was in the e.s.u.’s assigned to the intensities based on individual error models. The residual factors 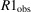 , 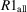 and 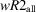 were calculated by *JANA2020* based on the common definitions:

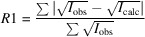




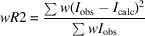




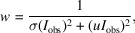

where *u* is the instability factor and the sum runs over all reflections in the case of 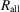 and 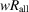 , and only over observed reflections with 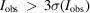 for the calculation of 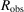 . 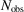 is the number of reflections with 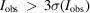 , 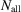 is the total number of reflections used in the refinement, 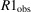 is the conventional *R* factor (*R*1) based on 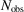 observed reflections, 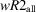 is the weighted *R* factor based on all reflections.

The application of the above weighting scheme is equivalent to changing the value of the coefficient 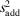 to 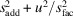 . With the default value of *u* used in *JANA2020* (*u* = 0.01, see Section 2) the change is negligible. We therefore decided to keep the default settings used in *JANA2020*.

The accuracy of the refined model is characterized by the RMSD of the covalent bond lengths for all non-H atoms from the respective reference values. Another assessment metric is based on the RMSD of atomic shifts of all non-H atoms from atom positions in the reference structures.

### 4.1. Refinement results

The kinematical refinement results are shown in Table 7 ▸ for natrolite, Table 8 ▸ for (*S*)-(+)-ibuprofen and Table 9 ▸ for l-alanine. These tables also contain the RMSD values of the refined covalent bond lengths for each model and of the distances of all non-H atoms from the positions in the reference structure. An important remark here is that the conventional 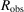 is not a particularly good measure of the refinement quality, as different error models result in a different number of observed reflections (see Section 5 for more discussion on observed reflections). 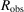 tends to increase with increasing 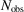 . A more robust way of assessing the different models and the quality of data in 3D ED is to compare the factors 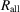 instead, which are directly comparable, as the complete set of intensities is the same for all models. Tables 7 ▸, 8 ▸ and 9 ▸ show that model 3 is the best error correction model for all tested data sets and across all comparison metrics, with a single exception of the RMSD of bond lengths for l-alanine, which is better for model 2 than model 3, but the difference is marginal.

#### 4.1.1. Natrolite

In the case of natrolite, model 1 using the standard deviation method for the error estimates introduces an evident improvement in the *R* factors and the goodness-of-fit parameter compared with the original model (Table 7 ▸). It further gives the best 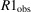 factor among all other models. However, as stated earlier, a good measure of comparison for the different techniques is 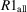 rather than 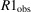 . The RMSDs of bond lengths and of the atomic shifts are better than those of the original model. Fig. 1 ▸(*b*) shows the relative improvement in the normal probability plot as well as the distribution of the adjusted normalized deviations compared with the model 0 [Fig. 1 ▸(*a*)].

As for model 2, in the case of natrolite, the values of the three correction parameters are: 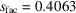 , 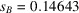 and 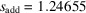 . From Table 7 ▸, it is obvious that there is a drop in 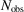 and a dramatic increase in 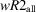 . This is the main drawback of this model. The refined structure model of natrolite is considerably improved upon applying the corrected errors of model 2 as can be seen from the RMSD values.

The values of the three correction parameters in the case of natrolite, model 3, are: 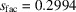 , 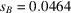 and 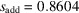 . Model 3 introduces an overall improvement of all the parameters. Firstly, the number of observed reflections is the highest of all models. As compared with model 2, this illustrates the benefit of using the largest intensity of the symmetry-related reflections for adjusting the error estimates, rather than the average intensity. This is also clear from the normal plots of natrolite (Fig. 1 ▸) where the normal plot of model 3 [Fig. 1 ▸(*d*)] is the most favourable. The distribution of the normalized deviations of natrolite in Fig. 1 ▸(*d*) also provides the best fit to a standard normal distribution. The data in this case have more positive deviations than negative deviations and this explains the slight shift of the histogram to the left [Fig. 1 ▸(*d*)]. The 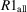 value is the best as well and it is 1.11 percentage points less than that of the original model. More importantly, the refined structure of natrolite corresponding to this model is the most accurate when compared with the reference model (Capitelli & Derebe, 2007 ▸), showing the best RMSD values of the bond lengths and the atomic positions of all non-H atoms. The kinematical refinement of model 3 of natrolite after the outlier rejection introduces a slight improvement in the 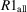 factor. The RMSD values for atomic shifts and bond lengths as shown in Table 10 ▸ are almost the same.

#### 4.1.2. Ibuprofen

In the case of (*S*)-(+)-ibuprofen, the situation is different regarding model 1. The latter does not present any improvement from the original model. On the contrary, the structure is slightly deformed. The refinement of the H atoms of hydroxyl groups was not stable. The aromatic carbon ring is also significantly distorted, leading to worse RMSD values as compared with the reference (King *et al.*, 2011 ▸). This is obvious from the distribution of data [Fig. 2 ▸(*b*)]. The two peaks at −0.707 and 0.707 indicate a high number of symmetry-related reflections (19%) that are measured only twice (*n* = 2).

Regarding the results of model 2 in the case of (*S*)-(+)-ibuprofen, the three correction parameters are: 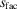 = 0.712995, 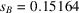 and 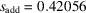 . 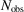 is reasonable for this data set and it is not reduced as compared with model 0. The normal plot in Fig. 2 ▸(*c*) shows a noticeable improvement from the original normal plot [Fig. 2 ▸(*a*)], but it is still not a perfectly fitting line. A positive and a negative tail are present, in addition to some other slight deviations from the theoretical line of slope 1. 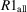 and the goodness-of-fit factors are enhanced, while 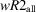 still records the highest value in this model. The structure model is better than the original one based on the RMSD values.

Finally, for model 3 in the case of (*S*)-(+)-ibuprofen, the three correction parameters are: 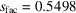 , 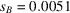 and 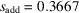 . Again, this model has the largest number of observed reflections. The 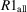 factor is reduced by 0.73 from that of the original model. The normal plot of the normalized deviations based on this model as shown in Fig. 2 ▸(*d*) is the nearest to normally distributed data. Fig. 2 ▸(*d*) also reveals the significance of this model in adjusting the sample data to better fit a standard Gaussian distribution. Additionally, the refined structure from model 3 is the most accurate among the others as indicated by the RMSD (Table 8 ▸). The kinematical refinement of model 3 after the outlier rejection in this case improves slightly the *R* factors (Table 10 ▸). The RMSDs for covalent bond lengths and atomic positions from the respective reference values improve as well after discarding the outliers.

#### 4.1.3. l-alanine

In the case of l-alanine, model 1 does not introduce any improvement of model 0. On the contrary, the value of the 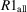 factor is increased, and the refined structure is deformed rather than enhanced by comparing the RMSD of bond lengths and atomic shifts of non-H atoms (Table 9 ▸).

As for model 2 in the case of l-alanine, the three correction parameters are: 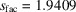 , 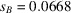 and 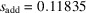 . The structure of this model has indeed the best value of RMSD of the bond lengths and the goodness-of-fit factor is enhanced, but the number of observed reflections is the lowest among the other models and the 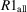 factor is also larger than that of model 0. This case again confirms the main drawbacks of this model. The normal plot in Fig. 3 ▸(*c*) shows a noticeable improvement from the original normal plot [Fig. 3 ▸(*a*)], but it is still not a perfectly fitting line. A recognizable positive tail is present in addition to some other slight deviations from the theoretical line of slope 1.

For model 3, this last data set again confirms that this is the best error correction model among the others. The 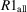 factor and the number of observed reflections are the best and, above all, the structure has the lowest RMSD of atomic shifts as compared with the reference (Parsons *et al.*, 2013 ▸). Fig. 3 ▸(*d*) shows a noticeable improvement in the normal probability plot and the distribution of the adjusted normalized deviations. The three correction parameters are now: 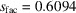 , 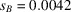 and 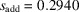 . The kinematical refinement results in the absence of outliers have indeed been improved as shown in Table 10 ▸. The 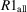 factor decreases from 14.73 to 14.16. The RMSDs for bond lengths and atomic shifts have been improved slightly after rejecting the 93 outliers.

## 5. Discussion

### 5.1. Observed reflections

**Figure 4:**
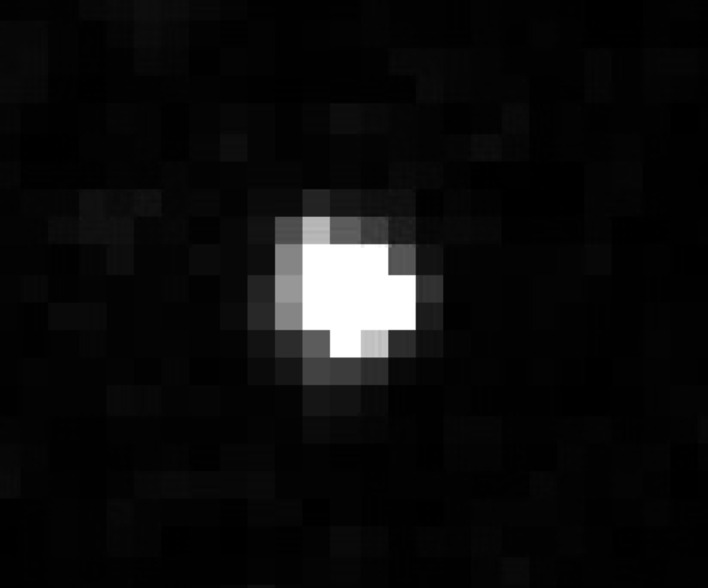
An image of a reflection (−1, −5, −9) from the diffraction pattern of the l-alanine data set of model 3 that is clearly visible, although it has *I* < 3σ(*I*) in the correction model 3.

It is customary to present refinement characteristics, typically the unweighted *R* value *R*1, calculated only on sufficiently strong reflections. A typical criterion is *I* > 3σ(*I*). The rationale behind this tradition is that weak reflections contain essentially only noise, and they do not provide useful information on the quality of the fit. The term used for reflections stronger than the selected criterion is ‘observed reflections’. The term ‘observed’ refers to the experiment, and to the fact that a reflection with *I* > 3σ(*I*) is usually visible in the diffraction pattern as a distinct intensity maximum, *i.e.* can be observed in the pattern. This is, however, meaningful only if σ(*I*) is calculated from counting statistics only. As soon as σ(*I*) is modified to account for other errors, the term ‘observed’ loses its original meaning. Specifically, when σ(*I*) is significantly increased due to the error-model correction, a reflection, which is clearly visible in the diffraction pattern, becomes formally ‘unobserved’, *i.e.* has *I* < 3σ(*I*). This is not a very big problem for typical X-ray diffraction data, where the corrections to the counting statistic σ(*I*) are generally small and affect mostly the strong reflections. However, it becomes a problem for data with dominant systematic errors, like those caused by the dynamical effects in 3D ED data. As an example, Fig. 4 ▸ shows an image of a reflection, which, after the error-model correction, has *I* = 1.85σ(*I*). Although the reflection is nominally unobserved, it is actually quite strong and clearly visible in the experimental data. The problem becomes even more serious when the ‘obs’ values are used for comparison between refinements. Different error models lead to different corrections to σ(*I*), hence to a different number of reflections with *I* > 3σ(*I*) and, as a consequence, to incomparable values of *R*(obs) and other obs-related statistics. As an example, error correction according to model 1 for natrolite yields 876 observed reflections with *I* > 3σ(*I*) and *R*1(obs) of 13.37%, while model 3 gives 1007 observed reflections (out of 1279) and *R*1(obs) of 14.67%. Thus, superficially, model 1 may appear to give a significantly better result, but it is just an artefact of the number of observed reflections. *R*1(all) as well as other statistics clearly show that the result from model 3 is superior.

One could thus conclude that the use of the term ‘observed reflection’ and related quantities is not meaningful for 3D ED data and should be discontinued. Other methods of estimating the amount of information present, *e.g.* the correlation-based techniques commonly used in macromolecular crystallography, may be more suitable. Until then, the scientists working with these data should be aware of the caveat just described, and use the term ‘observed reflections’ with caution and with awareness of its limitations.

### 5.2. Features of the error correction models

A thorough comparison of the refinements reveals that in all cases the set of adjustments propagated in error model 3 gives significantly improved results.

Model 1 did not present any improvement in the cases of (*S*)-(+)-ibuprofen and l-alanine. In the case of natrolite, the structure is slightly enhanced. Model 1 is expected to be more successful with data with a high multiplicity of symmetry-related reflections since the only information it is based on is the observed variation among these reflections. Stated differently, estimates of the individual errors of this model, derived only from the standard error of the mean of the reflections, become less adequate in the case of data with a low redundancy of symmetry-related reflections. This in turn may result in serious inaccuracy of the refined structure model. (*S*)-(+)-ibuprofen for instance has a monoclinic crystal system, and thus many reflections have a low redundancy of 2. This explains the significant improvement in the case of natrolite, which has an orthorhombic crystal system of higher redundancy. In the case of l-alanine, although it has an ortho­rhombic crystal system, many reflections of this data set (25%) have a multiplicity of *n* = 2 due to the low completeness of the data set. This explains the inefficiency of model 1 in this case.

Model 2 clearly provides an improvement, but it still has some deficiencies that need to be worked through. In some cases (natrolite and l-alanine) an obvious drop in 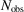 was evident. By looking at the correction parameters, we notice that in both cases one of these parameters is larger than 1 ( 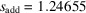 for natrolite and 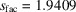 for l-alanine). This signifies a substantial inflation of error estimates. This inflation makes the model less reliable in many situations. In the case of ibuprofen, all the correction parameters are less than 1 and the inflation of the e.s.u.’s is not so dramatic. This is due to the fact that ibuprofen is monoclinic. The variations among the intensities of the symmetry-related reflections are less prominent. This means the values of the correction parameters are not so large and thus 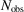 remains reasonable for this data set and does not need to be reduced.

In fact, the correction parameters 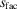 and 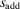 of a well processed data set should have their values close to 1 and 0, respectively. 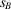 is usually negligible with a value close to 0. The exact values will certainly depend on the Laue class of the corresponding data set, the redundancy of the symmetry-related reflections and the variations among their intensities. It is worth noting that the three refined parameters of model 3 in general have lower values than their analogues of model 2 for all three samples. In model 3, less compensation is needed to correct for the gain detector uncertainty and other types of errors, owing to the use of the largest intensity of symmetry-related observations instead. Model 3 adjusts the error estimates of the strongest reflections without unnecessary exaggeration of the e.s.u.’s. It provides a good compromise between adjusting the error estimates and maintaining a decent number of observed reflections.

## 6. Conclusion

Various models for adjustment of estimated standard uncertainties of reflection intensities in 3D ED data were investigated with the aim of verifying if the models commonly used for single-crystal X-ray diffraction data are also suitable for 3D ED data. The tests on three experimental data sets showed that the best model is model 3, which differs from the commonly used approach by employing the maximum of the symmetry-equivalent intensities in the calculation of normalized residuals rather than the average value.

It is not surprising that accurate estimates of the e.s.u.’s are useful, but it is notable how much improvement model 3 brings to the kinematical refinement compared with the case with no error-model adjustment, but also with the other tested models. The benefits of using the model include an overall enhanced accuracy of atomic positions, covalent bond lengths and improved *R* factors. The benefits of model 3 are expected to be most pronounced in data with low redundancy and large variation in the intensities of symmetry-related reflections. It may be expected that with data obtained by averaging a large number of individual data sets, an approach becoming more and more popular in contemporary 3D ED studies, the differences between the models would become smaller.

The procedure according to model 3 is implemented in the software package *PETS2* (Palatinus *et al.*, 2019 ▸), available at http://pets.fzu.cz/.

## Supplementary Material

Crystal structure: contains datablock(s) global, ibuprofen_model0, ibuprofen_model1, ibuprofen_model2, ibuprofen_model3, ibuprofen_model3_outliers, L-alanine_model0, L-alanine_model1, L-alanine_model2, L-alanine_model3, L-alanine_model3_outliers, natrolite_model0, natrolite_model1, natrolite_model2, natrolite_model3, natrolite_model3_outliers. DOI: 10.1107/S2053273323005053/pl5027sup1.cif

Structure factors: contains datablock(s) ibuprofen_model0. DOI: 10.1107/S2053273323005053/pl5027ibuprofen_model0sup2.hkl

Structure factors: contains datablock(s) ibuprofen_model1. DOI: 10.1107/S2053273323005053/pl5027ibuprofen_model1sup3.hkl

Structure factors: contains datablock(s) ibuprofen_model2. DOI: 10.1107/S2053273323005053/pl5027ibuprofen_model2sup4.hkl

Structure factors: contains datablock(s) ibuprofen_model3. DOI: 10.1107/S2053273323005053/pl5027ibuprofen_model3sup5.hkl

Structure factors: contains datablock(s) L-alanine_model0. DOI: 10.1107/S2053273323005053/pl5027L-alanine_model0sup6.hkl

Structure factors: contains datablock(s) L-alanine_model1. DOI: 10.1107/S2053273323005053/pl5027L-alanine_model1sup7.hkl

Structure factors: contains datablock(s) L-alanine_model2. DOI: 10.1107/S2053273323005053/pl5027L-alanine_model2sup8.hkl

Structure factors: contains datablock(s) L-alanine_model3. DOI: 10.1107/S2053273323005053/pl5027L-alanine_model3sup9.hkl

Structure factors: contains datablock(s) natrolite_model0. DOI: 10.1107/S2053273323005053/pl5027natrolite_model0sup10.hkl

Structure factors: contains datablock(s) natrolite_model1. DOI: 10.1107/S2053273323005053/pl5027natrolite_model1sup11.hkl

Structure factors: contains datablock(s) natrolite_model2. DOI: 10.1107/S2053273323005053/pl5027natrolite_model2sup12.hkl

Structure factors: contains datablock(s) natrolite_model3. DOI: 10.1107/S2053273323005053/pl5027natrolite_model3sup13.hkl

Structure factors: contains datablock(s) I. DOI: 10.1107/S2053273323005053/pl5027ibuprofen_model3_outlierssup14.hkl

Structure factors: contains datablock(s) I. DOI: 10.1107/S2053273323005053/pl5027L-alanine_model3_outlierssup15.hkl

Structure factors: contains datablock(s) I. DOI: 10.1107/S2053273323005053/pl5027natrolite_model3_outlierssup16.hkl

CCDC references: 2268164, 2268165, 2268166, 2268167, 2268168, 2268169, 2268170, 2268171, 2268172, 2268173, 2268174, 2268175, 2268176, 2268177, 2268178

## Figures and Tables

**Table 1:** Measurement conditions of natrolite

Microscope	FEI Tecnai G2 20
Detector (type)	Olympus SIS Veleta (CCD)
3D ED data sets	One continuous rotation
λ (Å)	0.02508
*T* (K)	293
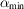 , 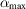 , Δα (°)	−50.0, 50.0, 0.6

**Table 2:** Sample overview of natrolite crystal

Empirical formula	Na_2_(Al_2_Si_3_O_10_)(H_2_O)_2_
*Z*	8
Crystal system	Orthorhombic
Space group	*Fdd*2
*a*, *b*, *c* (Å)	18.2872 (11), 18.6661 (14), 6.6222 (3)
α, β, γ (°)	90, 90, 90
*V* (Å^3^)	2260.5 (2)
d*max (Å^−1^)	1.6
*R*_int_(all)	19.80%
Mosaicity (°)	0.15
Completeness	99%

**Table 3:** Measurement conditions of (*S*)-(+)-ibuprofen

Microscope	FEI Tecnai G2 20
Detector (type)	ASI Cheetah
3D ED data sets	Two continuous rotations
λ (Å)	0.02508
*T* (K)	95.15
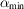 , 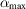 , Δα (°) #1	−55.0, 30.0, 0.25
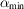 , 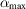 , Δα (°) #2	−50.0, 40.0, 0.25

**Table 4:** Sample overview of (*S*)-(+)-ibuprofen crystal

Empirical formula	C_13_H_18_O_2_
*Z*	4
Crystal system	Monoclinic
Space group	*P*2_1_
*a*, *b*, *c* (Å)	12.368 (4), 8.021 (3), 13.536 (5)
α, β, γ (°)	90, 112.24 (3), 90
*V* (Å^3^)	1242.9 (8)
d*max (Å^−1^)	1.0
*R*_int_(all)	16.59%
Mosaicity (°)	0.135
Completeness	85%

**Table 5:** Measurement conditions of L-alanine

Microscope	FEI Tecnai G2 20
Detector (type)	ASI Cheetah
3D ED data sets	One continuous rotation
λ (Å)	0.02508
*T* (K)	100
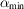 , 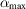 , Δα (°)	−40.0, 40.0, 0.5

**Table 6:** Sample overview of L-alanine crystal

Empirical formula	C_3_H_7_NO_2_
*Z*	4
Crystal system	Orthorhombic
Space group	*P*2_1_2_1_2_1_
*a*, *b*, *c* (Å)	5.7733 (11), 5.9524 (12), 12.247 (2)
α, β, γ (°)	90, 90, 90
*V* (Å^3^)	420.85 (14)
d*max (Å^−1^)	2.0
*R*_int_(all)	11.11%
Mosaicity (°)	0.3
Completeness	56%

**Table 7:** Kinematical refinement results of natrolite for the different error estimate models (*N*_par_ is the number of refinement parameters)

Natrolite	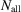	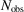	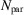	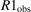 (%)	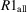 (%)	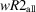 (%)	GOF(all)	RMSD of bond lengths (Å)	RMSD of atomic shifts (Å)
Original	1289	839	93	14.58	17.20	30.52	6.26	0.0211	0.0310
Model 1	1289	876	93	13.37	16.88	26.42	2.93	0.0194	0.0247
Model 2	1289	801	93	14.90	16.48	40.60	1.48	0.0120	0.0174
Model 3	1289	1007	93	14.67	16.09	36.34	1.78	0.0092	0.0151

**Table 8:** Kinematical refinement results of (*S*)-(+)-ibuprofen for the different error estimate models

(*S*)-(+)-ibuprofen	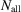	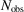	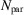	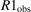 (%)	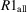 (%)	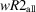 (%)	GOF(all)	RMSD of bond lengths (Å)	RMSD of atomic shifts (Å)
Original	1212	825	127	17.94	21.93	31.91	4.41	0.0711	0.1113
Model 1	1212	856	127	19.11	23.36	40.71	5.46	0.0786	0.1121
Model 2	1212	883	127	18.82	21.45	42.60	2.03	0.0547	0.0702
Model 3	1212	957	127	19.01	21.20	40.97	2.36	0.0498	0.0665

**Table 9:** Kinematical refinement results of L-alanine for the different error estimate models

L-alanine	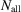	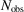	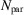	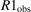 (%)	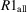 (%)	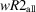 (%)	GOF(all)	RMSD of bond lengths (Å)	RMSD of atomic shifts (Å)
Original	1127	1036	56	14.28	14.82	34.80	8.76	0.0143	0.0244
Model 1	1127	922	56	13.92	15.71	33.76	5.35	0.0194	0.0290
Model 2	1127	845	56	13.95	15.31	34.47	2.03	0.0070	0.0210
Model 3	1127	1072	56	14.44	14.73	35.51	2.57	0.0072	0.0195

**Table 10:** Kinematical refinement results of the three data sets after the outlier rejection corresponding to model 3

	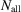	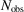	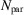	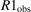 (%)	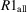 (%)	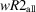 (%)	GOF(all)	RMSD of bond lengths (Å)	RMSD of atomic shifts (Å)
Natrolite	1289	1008	93	14.69	16.06	36.60	1.78	0.0093	0.0151
(*S*)-(+)-ibuprofen	1209	972	127	19.02	21.10	41.39	2.35	0.0489	0.0656
L-alanine	1123	927	56	13.06	14.16	30.49	2.36	0.0067	0.0193
